# Vertical Transmission of the Zika Virus Causes Neurological Disorders in Mouse Offspring

**DOI:** 10.1038/s41598-018-21894-w

**Published:** 2018-02-23

**Authors:** Yingchao Shi, Shihua Li, Qian Wu, Le Sun, Junjing Zhang, Na Pan, Qihui Wang, Yuhai Bi, Jing An, Xuancheng Lu, George Fu Gao, Xiaoqun Wang

**Affiliations:** 1 State Key Laboratory of Brain and Cognitive Science, CAS Center for Excellence in Brain Science and Intelligence Technology (Shanghai), Institute of Biophysics, Chinese Academy of Sciences, Beijing, 100101 China; 2 CAS Key Laboratory of Pathogenic Microbiology & Immunology, Institute of Microbiology, Chinese Academy of Sciences, Beijing, 100101 China; 3 University of Chinese Academy of Sciences, Beijing, 100049 China; 4 Beijing Institute for Brain Disorders, Beijing, 100069 China; 5 Savaid Medical School, University of Chinese Academy of Sciences, Beijing, 100049 China; 6 National Institute for Viral Disease Control and Prevention, Chinese Center for Disease Control and Prevention, Beijing, 102206 China; 7 Department of Microbiology, School of Basic Medical Sciences, Capital Medical University, Beijing, 100069 China; 8 Research Network of Immunity and Health, Beijing Institutes of Life Science, Chinese Academy of Sciences, Beijing, 100101 China; 9 Laboratory Animal Center, Chinese Center for Disease Control and Prevention, Beijing, 102206 China

## Abstract

The association between Zika virus (ZIKV) infection and congenital malformations such as microcephaly in infants is a public health emergency. Although various *in vivo* and *in vitro* models are used for ZIKV research, few animal models are available for resolving the effects of maternal ZIKV infection on neonatal development. Here, we established an immunocompetent mouse model via intrauterine inoculation. Our results confirmed that ZIKV, but not dengue virus, infection caused spontaneous abortions, brain malformations, ocular abnormalities, spinal cord defects and paralysis in mouse offspring. Aside from microcephaly and hippocampal dysplasia, eye abnormalities, including microphthalmia, thinner optic nerves, damaged retinae, and deficient visual projection, were also observed following ZIKV infection. Moreover, ZIKV-infected offspring showed a loss of alpha motor neurons in the spinal cord and cerebellar malformation, which may cause paralysis. ZIKV also impaired adult neurogenesis in neonatal mice. Due to its intact immunity, our rodent model can be used to systematically evaluate the impact of ZIKV on embryonic and neonatal development and to explore potential therapies.

## Introduction

Zika virus (ZIKV) is an emerging, positive-stranded RNA arbovirus that, together with several other pathogens, such as dengue virus (DENV), yellow fever virus (YFV), West Nile virus (WNV), Japanese encephalitis virus (JEV), and tick-borne encephalitis virus (TBEV), belongs to the *Flaviviridae* family^1^. As an arbovirus, apart from common transmission via mosquito bites^2,3^, ZIKV can also be passed from mothers to fetuses during pregnancy^4^, transmitted by sexual activities^5^, or acquired via blood transfusions in humans^6^.

ZIKV infection is generally believed to only cause mild clinical syndromes in adults^7^. However, a recent outbreak of ZIKV infection in Brazil was associated with an increase in pregnant women giving birth to microcephalic babies^8^. ZIKV was detected in the placenta and amniotic fluid of pregnant women with microcephalic fetuses, as well as in the blood of microcephalic newborns^9,10^. This ZIKV outbreak showed that ZIKV could cause severe clinical consequences, including congenital malformations such as spontaneous abortion, microcephaly and intrauterine growth restriction (IUGR) in infants^11,12^ and Guillain-Barré syndrome (GBS) in adults^13,14^.

The emerging association between ZIKV infection of pregnant women and fetal congenital abnormalities highlights the necessity for experimental systems to model ZIKV infection, probe pathological changes, look for potential treatments, and validate the effects of ZIKV in human clinical observations. Various *in vivo* and *in vitro* models have been established for ZIKV research. Due to the ethical regulation of human samples, neurospheres and brain organoids are used as complementary models for studying the effects of ZIKV infection on embryonic brain development *in vitro*^15^. Tang, *et al*. reported that, *in vitro*, ZIKV directly infects human cortical neural progenitor cells with high efficiency, leading to cell death and cell-cycle dysregulation^16^. Analogously, *in vitro* infection of human neurosphere organoid cultures with ZIKV impaired cell growth and increased cell death^17^. The detrimental effects of ZIKV on progenitor cells may explain why ZIKV causes microcephaly. Indeed, intraventricular inoculation of ZIKV into the fetuses of wild-type mice resulted in progenitor cell-specific ZIKV infection, cortical thinning, and microcephaly^18^.

Aside from brain hypoplasia, eye abnormalities such as microphthalmia, retinal pigmentary changes, chorioretinal atrophy, vasculature changes, and optic nerve hypoplasia have also been observed in the neonates of mothers infected with ZIKV during pregnancy^19–23^. Although ZIKV directly injected into the eyes of C57BL/6 mice^24^ or subcutaneously inoculated into *Ifnar1*^−/−^ mice with compromised immunity^25^ can cause ocular pathology, such as conjunctivitis or panuveitis in adult mice, no animal models are available for investigating the effects of maternal ZIKV infection on the ophthalmic development of offspring. Furthermore, studies on the effects of maternal ZIKV infection on offspring development mainly depend on suppressing the production or response of type I interferon (IFN) (e.g., *Ifnar1*^−/−^ mice)^26,27^, which might not reflect the truth. Although intrauterine or intravaginal ZIKV infection of immunocompetent, pregnant mice was found to cause placental dysfunction, fetal growth restriction, and abnormal fetal brain development^27,28^, a high dose of the virus was used. Furthermore, the developmental defects in the offspring have not been systemically analyzed. Hence, establishing proper immunocompetent animal models to mimic the natural vertical transmission of ZIKV is needed to study ZIKV tropism and neuropathogenesis in fetal CNS development.

To evaluate the relationship between maternal ZIKV infection and developmental defects in offspring, we established an experimental immunocompetent rodent model. We intrauterinely inoculated C57BL/6 timed-pregnant mice with ZIKV on embryonic day 13.5 (E13.5). We found that intrauterine ZIKV inoculation led to spontaneous abortions, brain malformations, ocular abnormalities, spinal cord defects, and paralysis in mouse offspring. This result is consistent with the neuropathological changes induced by congenital ZIKV infection in humans. In addition to microcephaly and an abnormal hippocampus, ZIKV-infected infant mice exhibited smaller eyeballs, thinner optic nerves, lesioned retinae, and impaired visual projection. Moreover, ZIKV-infected offspring displayed a loss of alpha motor neurons in the spinal cord and cerebellar malformation, which may cause paralysis. We also confirmed adult neurogenesis impairment of ZIKV-infected offspring. These defects were not observed in the offspring of DENV-2-inoculated pregnant mice, which suggests the neurotropic specificity of ZIKV infection. Due to its innate immunity, our rodent model can be implemented to systemically evaluate the pathological effects of ZIKV on human fetal development and explore potential therapies.

## Results

### A model for ZIKV vertical transmission

**Figure 1:**
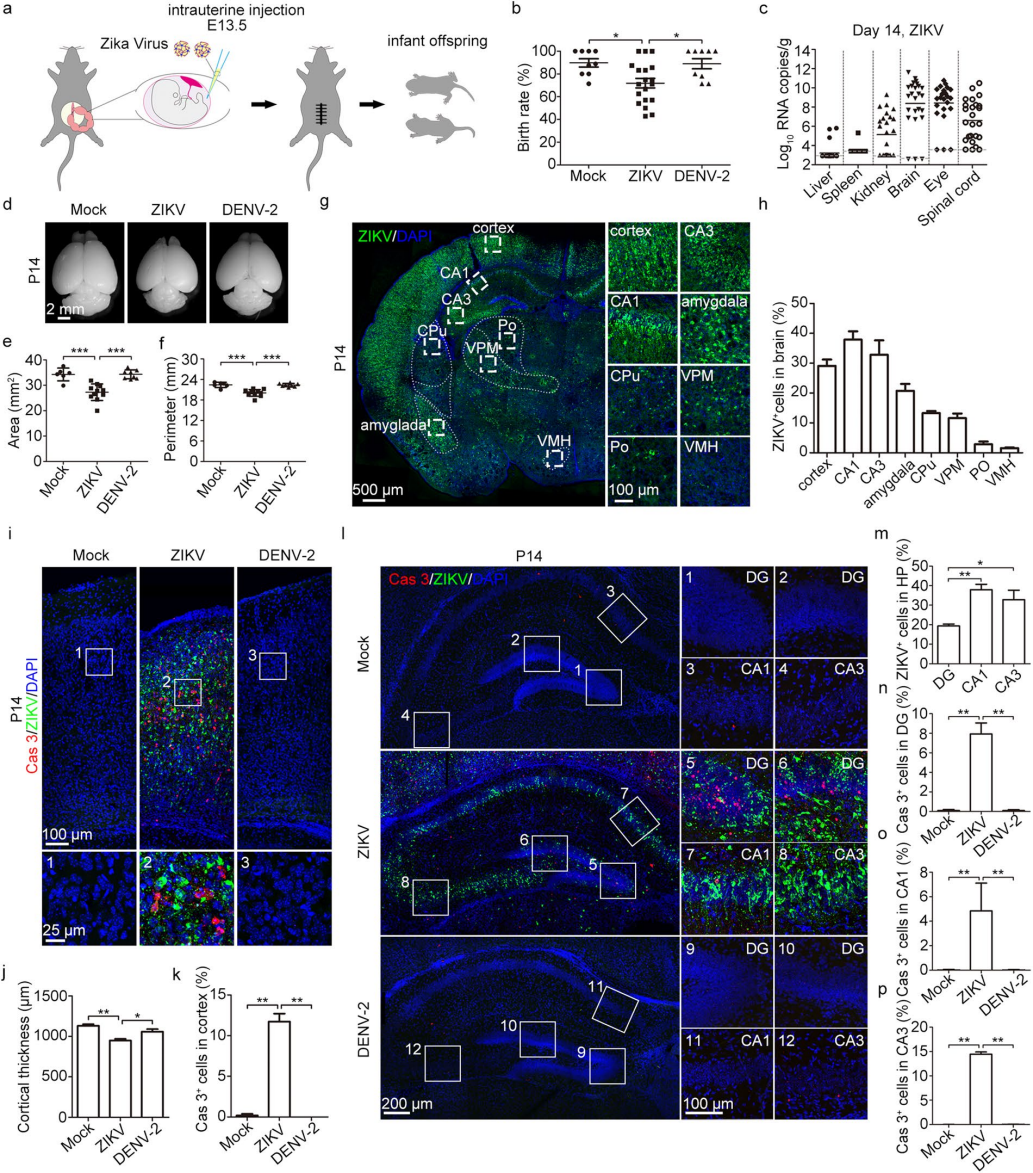
Low birth rate, high mortality and severe brain pathology induced by intrauterine ZIKV inoculation. (a) Schematic depiction of intrauterine inoculation during pregnancy. Offspring mice were used in following studies. (b) Birth rate of neonatal mice. (c) ZIKV viral load in P14 tissues was measured by qRT-PCR. Bars indicate the mean. The gray dashed lines indicate the limit of detection. (d) Representative images of brains from P14 offspring. (e,f) Area (e) and perimeter (f) of the hemiencephalon of P14 mice. (g) Overview of ZIKV infection in 1 representative brain slice. CPu, caudate putamen (striatum); Po, posterior thalamic nuclear group; VPM, ventral posteromedial thalamic nucleus; VMH, ventromedial hypothalamic nucleus. (h) Percentage of ZIKV-infected cells in different brain regions. n = 5 samples from three independent experiments. (i) Representative images of the cortex stained for Cas3 (Caspase 3) and ZIKV. (j) Quantification of cortical thickness. (k) Percentage of Cas3-positive cells within the cortex. (j,k) n = 5 samples from three independent experiments. (l) Representative images of the hippocampus stained for Cas3 and ZIKV. (m) Percentage of ZIKV-infected cells in the DG, CA1, and CA3. n = 5 samples from three independent experiments. (n–p) Percentage of Cas3-positive cells within the DG(n), CA1(o), and CA3(p), respectively. n^Mock^ = 4, n^ZIKV^ = 5, n^DENV-2^ = 4 samples from three independent experiments. Quantification data are presented as the mean ± SEM. One-way ANOVA with Bonferroni’s post hoc test (*p < 0.05, **p < 0.01, ***p < 0.001).

To evaluate whether intrauterine ZIKV inoculation during pregnancy could cause ZIKV infection in mouse offspring, pregnant immunocompetent C57BL/6 mice were used. To determine whether the developmental defects observed in infected offspring are ZIKV-specific or common to flaviviruses, DENV-2 (a flavivirus similar to ZIKV) was used as a viral control in our study. ZIKV (SMGC-1)^29^ or DENV-2 (dengue virus serotype 2, strain 43) were injected directly into the amniotic fluid of C57BL/6 timed-pregnant mice on E13.5, at a dose of 1500 plaque-forming units (PFU)/fetus (Fig. 1a). Intrauterine injection of DMEM (Dulbecco’s modified Eagle’s medium, virus culture medium) was used as a blank control (hereafter denoted as, “mock”). On the day of birth, the birth rate of the mice was analyzed. Compared with mock- and DENV-2-infected mice, the birth rate of ZIKV-infected neonatal mice was significantly reduced (71.9%) (Fig. 1b, n = 19 litters). This result was consistent with the high rate of spontaneous abortions observed in ZIKV-infected pregnant women^7^. To determine whether offspring mice could be effectively infected, tissues from infant mice were collected at postnatal days P0, P7, P14, P21, and P28. The viral loads were measured by quantitative real-time RT-PCR (qRT-PCR) (Fig. 1c and Supplementary Fig. S1a–e). ZIKV infection efficiency was evaluated as 88.6% from samples obtained at P7 (n = 59, 10 complete litters with some individuals from other litters). ZIKV RNAs were detectable in the kidneys, eyes, and spinal cords of some offspring at P0 (Supplementary Fig. S1a), and ZIKV RNAs reached peak levels at P7 (Supplementary Fig. S1b). High levels of viral RNA persisted up to P14, but dropped at P21 and P28 (Fig. 1c and Supplementary Fig. S1c,d). The tissue preference is in accordance with previous observations indicating that ZIKV infection is neurotropic^30^. In contrast, DENV-2 RNAs were not detectable in any tissues of P0 newborn mice. Small amounts of DENV-2 RNAs could be detected in some tissue samples of a few animals at P7. However, these DENV-2 RNAs became undetectable at P14 (Supplementary Fig. S1e). In line with the ZIKV-related fetal death observed in humans^7^, 74.3% of ZIKV-infected offspring mice died before P28 (Supplementary Fig. S1f, n = 19 litters). In summary, intrauterine ZIKV inoculation is a practical method for establishing a valid immunocompetent animal model to assess how maternal ZIKV infection during pregnancy influences the development of embryos and infants.

To examine how mother-to-fetus vertical ZIKV transmission causes pathological changes in offspring in the context of competent immune responses, we first evaluated the effects of ZIKV infection on brain. In pregnant women, ZIKV infection caused catastrophic neurologic complications, such as microcephaly^7^. Likewise, smaller brain sizes were observed in ZIKV-infected P14 infant mice, as indicated by the reduced area and perimeter of the hemiencephalon (Fig. 1d–f). To further determine the infected tissues, viral protein expression was detected by immunofluorescence assay with monoclonal antibody Z6^31^. In P14 mice, robust ZIKV E protein expression was observed in the cortex, hippocampus (CA1 and CA3 regions), and amygdala, while weak expression was detected in the striatum, thalamus, and hypothalamus (Fig. 1g–h). The Z6 mAb was previously shown to have cross-reactivity against DENV-2 E protein and has been used for DENV-2 detection^29^. However, immunostaining did not indicate viral infection of the DENV-2 intrauterine-inoculated offspring (Supplementary Fig. S1g). Following intrauterine ZIKV infection, 29.1% of cells in the infant cortex were infected by ZIKV and most were positive for the activated form of Caspase-3 (Fig. 1i–k). These data indicate that maternal ZIKV infection may induce severe cell apoptosis in the cortex of offspring, thus resulting in microcephaly. In the hippocampus, ZIKV-infected cells were highly represented in the DG, CA1, and CA3 regions (Fig. 1l,m). Compared to mock- and DENV-2-infected brains, the percentage of Caspase-3-positive cells in the DG, CA1, and CA3 regions of the hippocampus of ZIKV-infected individuals were significantly higher (Fig. 1l,n–p). This suggests that maternal ZIKV infection during pregnancy results in robust ZIKV infection and cell apoptosis in multiple regions of brain, which may contribute to microcephaly and brain dysfunction as infants mature.

### ZIKV causes visual system defects

**Figure 2:**
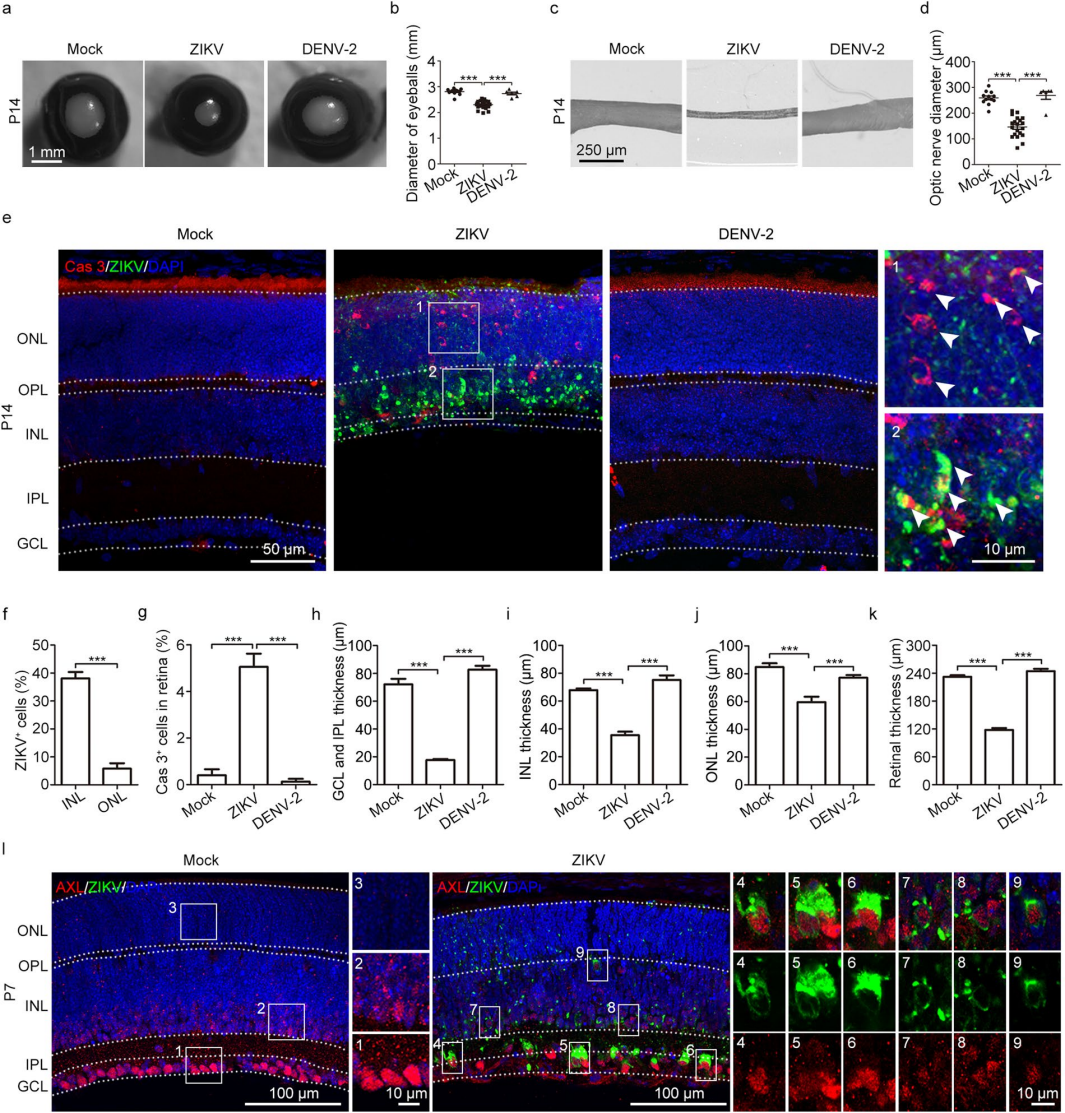
Visual defects caused by maternal ZIKV infection. (a) Representative images of P14 eyeballs. (b) Diameters of P14 eyeballs. (c) Representative images of P14 optic nerves. (d) Diameters of P14 optic nerves. (e) Representative images of the retina stained for Cas3 and ZIKV. White arrows, ZIKV-positive or Cas3-positive cells. (f) Percentage of ZIKV-infected cells within the INL and ONL. n = 10 samples from three independent experiments. (g) Percentage of Cas3-positive cells within the retina. n^Mock^ = 5, n^ZIKV^ = 9, n^DENV-2^ = 5 samples from three independent experiments. (h–j) Retinal GCL and IPL thickness (h), INL thickness (i), and ONL thickness (j), respectively. n^Mock^ = 14, n^ZIKV^ = 13, n^DENV-2^ = 11 samples from three independent experiments. (k) Quantification of retinal thickness. n^Mock^ = 14, n^ZIKV^ = 19, n^DENV-2^ = 11 samples from three independent experiments. (l) AXL and ZIKV expression in the P7 retina. Quantification data are presented as the mean ± SEM. Student’s *t*-test (f) or one-way ANOVA with Bonferroni’s post hoc test, ***p < 0.001.

Apart from the reported syndromes of microcephaly, IUGR, and fetal demise, several clinical studies have observed eye abnormalities in neonates born to mothers infected with ZIKV during pregnancy^19,22,23,32,33^. Compared with mock- and DENV-2-infected mouse offspring, smaller eyeballs and thinner optic nerves were observed in ZIKV-infected infant mice at P7 and P14, as indicated by the significantly decreased diameters of the eyeballs and optic nerves (Fig. 2a–d and Supplementary Fig. S2a–d). ZIKV infection was observed in the retinae of P7 and P14 mice, whereas DENV-2 caused no infection or defects (Fig. 2e and Supplementary Fig. S2e). At P7, the retinal ZIKV infection was preferentially distributed in the ganglion cell layer (GCL) and inner nuclear layer (INL); most of the ZIKV-infected cells were also Caspase-3-positive (Supplementary Fig. S2e). At P14, the retinal infection mainly targeted cells in the INL and outer nuclear layer (ONL) and displayed striking Caspase-3 staining; the GCL nearly disappeared at this time point (Fig. 2e–g). Furthermore, compared to mock- and DENV-2-infection, ZIKV infection brought about visible changes in the retinal cytoarchitecture. Briefly, the GCL, inner plexiform layer (IPL), INL, and ONL became extremely thin, while the outer plexiform layer (OPL) completely vanished. These changes explain the significantly decreased retinal thickness (Fig. 2h–k). The severe cell apoptosis induced by ZIKV infection may account for the changes in the retinal cytoarchitecture. Since AXL is speculated to be a candidate ZIKV entry cofactor/attachment receptor^34–37^, we performed immunofluorescence staining of AXL in P7 mice retinae. We observed that AXL was highly expressed in GCL cells and mildly expressed in INL cells (Fig. 2l). In the P7 mouse retinae, the AXL-specific expression pattern was coincident with the retinal cell tropism of ZIKV infection.

**Figure 3:**
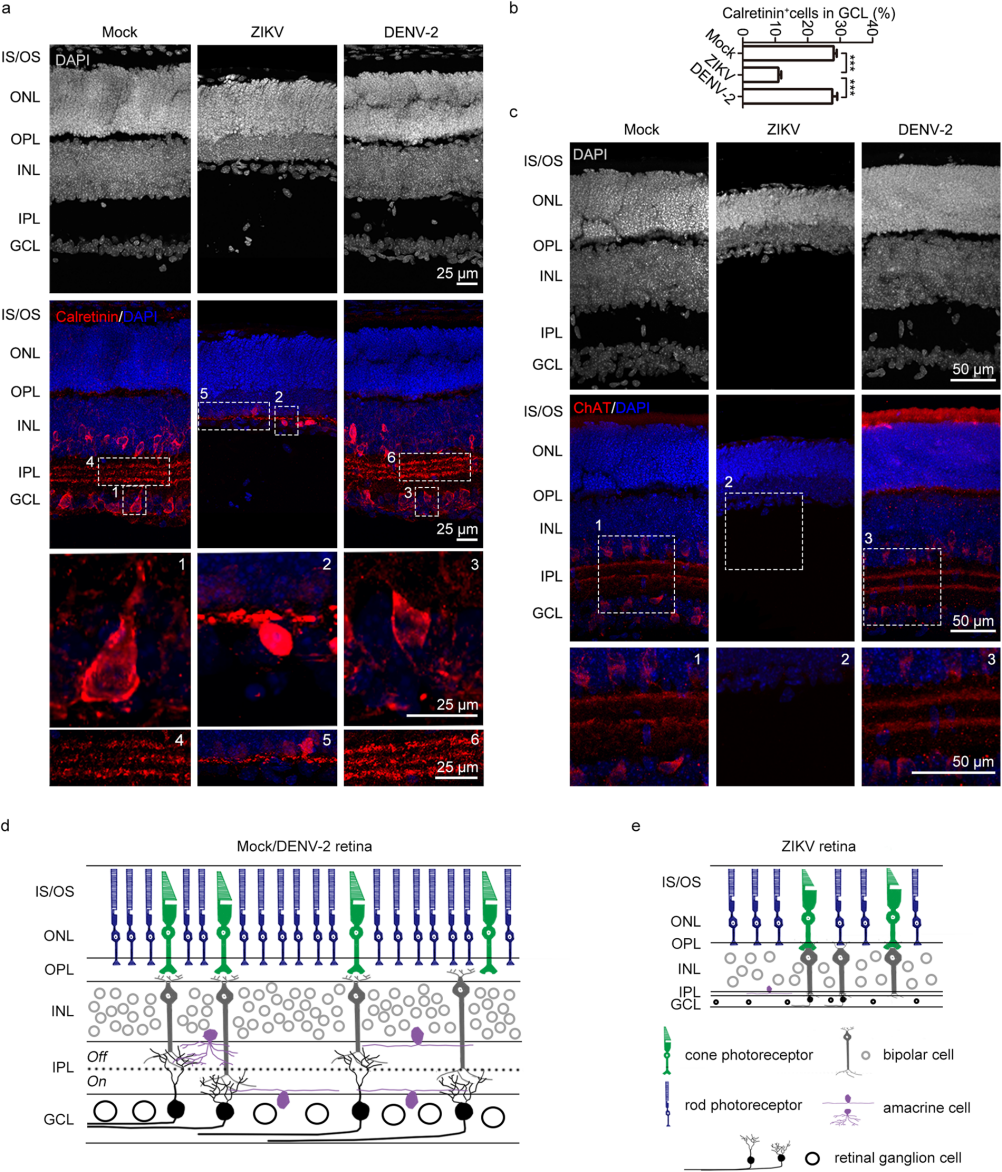
Few retinal ganglion cells and no starburst amacrine cells were left after maternal ZIKV infection. (a) P14 retinal Calretinin immunostaining. (b) Percentage of Calretinin-positive cells in the GCL. n^Mock^ = 7, n^ZIKV^ = 5, n^DENV-2^ = 8 samples from three independent experiments. Data are presented as the mean ± SEM. One-way ANOVA with Bonferroni’s post hoc test, ***p < 0.001. (c) P14 retinal ChAT immunostaining. (d,e) Schematic depiction of a normal retina in mock- or DENV-2 infected mice (d) or a pathological retina in ZIKV-infected mice (e).

To explore what types of retinal cells were reduced in ZIKV-infected mice, the retinal structure of P14 mice was further analyzed when severe atrophy was detected. It is known that synapses are located at the OPL and IPL^38^. In P14 ZIKV-infected retinae, we observed that the OPL was completely missing, while the IPL was extremely thin (Fig. 2e and 3a), suggesting a disruption of synaptic connections in the retinae. Given that the optic nerves of ZIKV-infected mice were thinner and the optic nerve consists of retinal ganglion cell axons^38^, we next investigated the effects of maternal ZIKV infection on the retinal ganglion cells of offspring. Calretinin, a calcium binding protein, was used as a specific marker of retinal ganglion and amacrine cells. After ZIKV infection, only a few Calretinin-positive cells with abnormal morphology remained in the GCL at P14 (Fig. 3a,b and Supplementary Fig. S3), indicating extensive cell death of the retinal ganglion cells. ZIKV-induced retinal ganglion cell death may contribute to optic nerve lesions. In contrast, these phenotypes were not observed in DENV-2-infected mice.

One specific type of amacrine cells, termed starburst amacrine cells, are essential for directionally selective retinal circuits^39^. To evaluate the effects of maternal ZIKV infection on the offspring’s starburst amacrine cells, immunostaining of acetylcholine (ChAT) was used to visualize starburst amacrine cells. The retinal ChAT immunostaining results showed that ChAT-positive cells were completely absent from the retinae of ZIKV-infected mouse offspring (Fig. 3c). This indicates a functional abnormality in the directionally selective retinal circuits. Thus, we developed a model of how maternal ZIKV infection during pregnancy affects offspring retinal development. Due to vertical ZIKV transmission, the total retinal thickness of the offspring was significantly reduced. The thickness of the GCL, IPL, INL, and ONL were significantly decreased, while the OPL had completely vanished. In addition, the majority of Calretinin-positive ganglion cells were absent, which may account for the pathology of the thin GCL and fine optic nerve. Furthermore, starburst amacrine cells, an essential element of directionally selective retinal circuits, were completely absent in infants with ZIKV infection (Fig. 3d,e).

**Figure 4:**
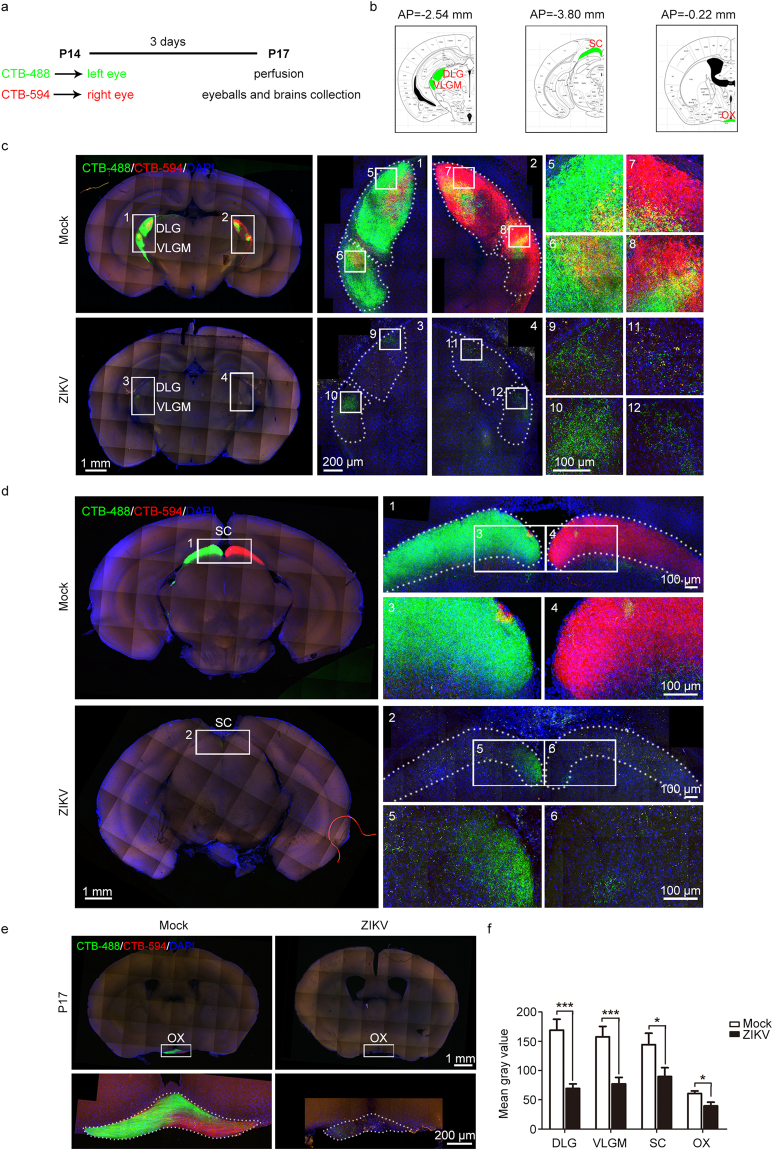
Impaired visual projection was induced by maternal ZIKV infection. (a) Schematic depiction of the anterograde tracing method used. (b) Brain atlases indicate the sites of brain slices in (c), (d), and (e), respectively. (c–e) Results of visual projection in the LGN (c), SC (d), and OX (e), respectively. (f) Mean gray value of visual projection fibers in the DLG, VLGM, SC, and OX. DLG and VLGM: n^Mock^ = 10, n^ZIKV^ = 14 samples; SC: n^Mock^ = 11, n^ZIKV^ = 10 samples; OX: n^Mock^ = 11, n^ZIKV^ = 9 samples from three independent experiments. Data are presented as the mean ± SEM. Student’s *t*-test, *p < 0.05, ***p < 0.001.

The reduced retinal ganglion cells and thinner optic nerves compelled us to explore whether visual projection via the optic nerves was also impacted by ZIKV infection. Anterograde tracing was performed via intraocular injection of cholera toxin B-Alexa Fluor 488 (CTB-488) or Alexa Fluor 594 (CTB-594) into either eye at P14. Three days later, the brains and eyeballs were collected (Fig. 4a). To ensure the reliability of the visual projection results, only mice with retinae homogeneously stained by CTB-488 or CTB-594 were used in the subsequent work (Supplementary Fig. S4). We mainly focused on the visual projections of the retina to the lateral geniculate nucleus (LGN), superior colliculus (SC), and optic chiasm (OX) (Fig. 4b–e). The visual projection fibers in the dorsal lateral geniculate nucleus (DLG) and ventral lateral geniculate nucleus pars medialis (VLGM) were reduced to 40%-50% of those in mock-infected mice (Fig. 4c and f). This indicates severe impairment of the retinal LGN projections. Unsurprisingly, sparse visual projection fibers were also observed in the SC and OX of ZIKV-infected offspring (Fig. 4d–f). Therefore, in accordance with thinner optic nerves, the visual projection observed in different brain areas was also significantly impaired following ZIKV infection, implying visual deficiencies in infant mice born from ZIKV-infected mothers.

### Paralysis and pathological changes in offspring

**Figure 5:**
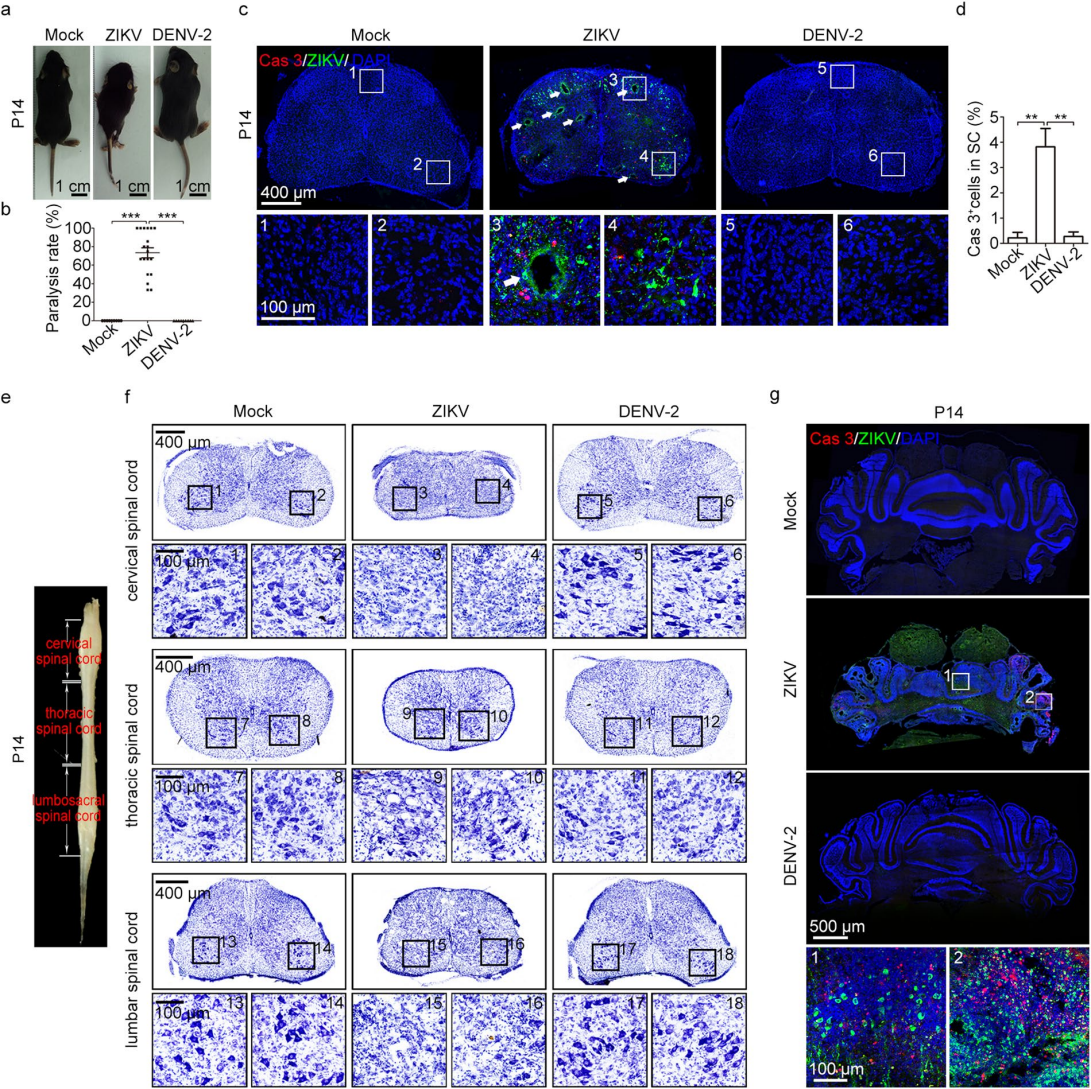
Severe movement defects were induced by maternal ZIKV infection. (a) Representative images of P14 offspring mice. The hind limbs of ZIKV-infected mice were paralyzed. (b) Quantification of the paralysis rate during P0-P28. (c) Representative images of lumbar spinal cords stained for Cas3 and ZIKV. White arrows, cavities. (d) Percentage of Cas3-positive cells within lumbar spinal cords. n^Mock^ = 4, n^ZIKV^ = 8, n^DENV-2^ = 4 samples from three independent experiments. (e) Representative image of a P14 spinal cord to illustrate the different segments. (f) Nissl staining of P14 cervical, thoracic, and lumbar spinal cord. (g) Representative images of the cerebellum stained for Cas3 and ZIKV. Quantification data are presented as the mean ± SEM. One-way ANOVA with Bonferroni’s post hoc test, **p < 0.01, ***p < 0.001.

We observed motor deficits in ZIKV-infected, but not mock- and DENV-2-infected, infant offspring (Fig. 5a). The hind limb paralysis rate in ZIKV-infected offspring mice was 73.4%, while no paralysis was observed in either mock- or DENV-2-infected offspring (Fig. 5b and Movie S1–3). Paralysis mainly occurred during P7-P14 (70.5%, n = 31/44). In the lumbar spinal cords of ZIKV-infected offspring at P14, we observed not only viral infection but also cell apoptosis (Fig. 5c,d). Many conspicuous cavities, possibly resulting from cell apoptosis, were present in the lumbar spinal cords of the ZIKV-infected mice (Fig. 5c). Thus, maternal ZIKV infection brought about severe spinal cord lesions in offspring mice. Alpha motor neurons, located at the anterior horn of the spinal cord, directly innervate skeletal muscle fibers^40^. Nissl staining results from the cervical, thoracic, and lumbar spinal cord showed that the majority of alpha motor neurons vanished after ZIKV infection. The remaining few alpha motor neurons exhibited abnormal morphology (Fig. 5e,f). Due to cell death, the thickness of the cervical, thoracic, and lumbar spinal cords dramatically decreased (Supplementary Fig. S5a–c). Additionally, we noticed robust viral protein expression and cell apoptosis in the cerebellum of offspring following maternal ZIKV infection, which may explain the cavities and reduced size of cerebellum (Fig. 5g and Supplementary Fig. S5d). These phenomena were not observed in the offspring of either mock- or DENV-2-infected pregnant mice (Fig. 5g and Supplementary Fig. S5d). Given that the cerebellum is essential for coordinating muscle movements and maintaining balance, the pathological phenotype in cerebellum may account, in part, for the movement deficiency of ZIKV-infected mice.

### ZIKV impairs adult neurogenesis in offspring

**Figure 6:**
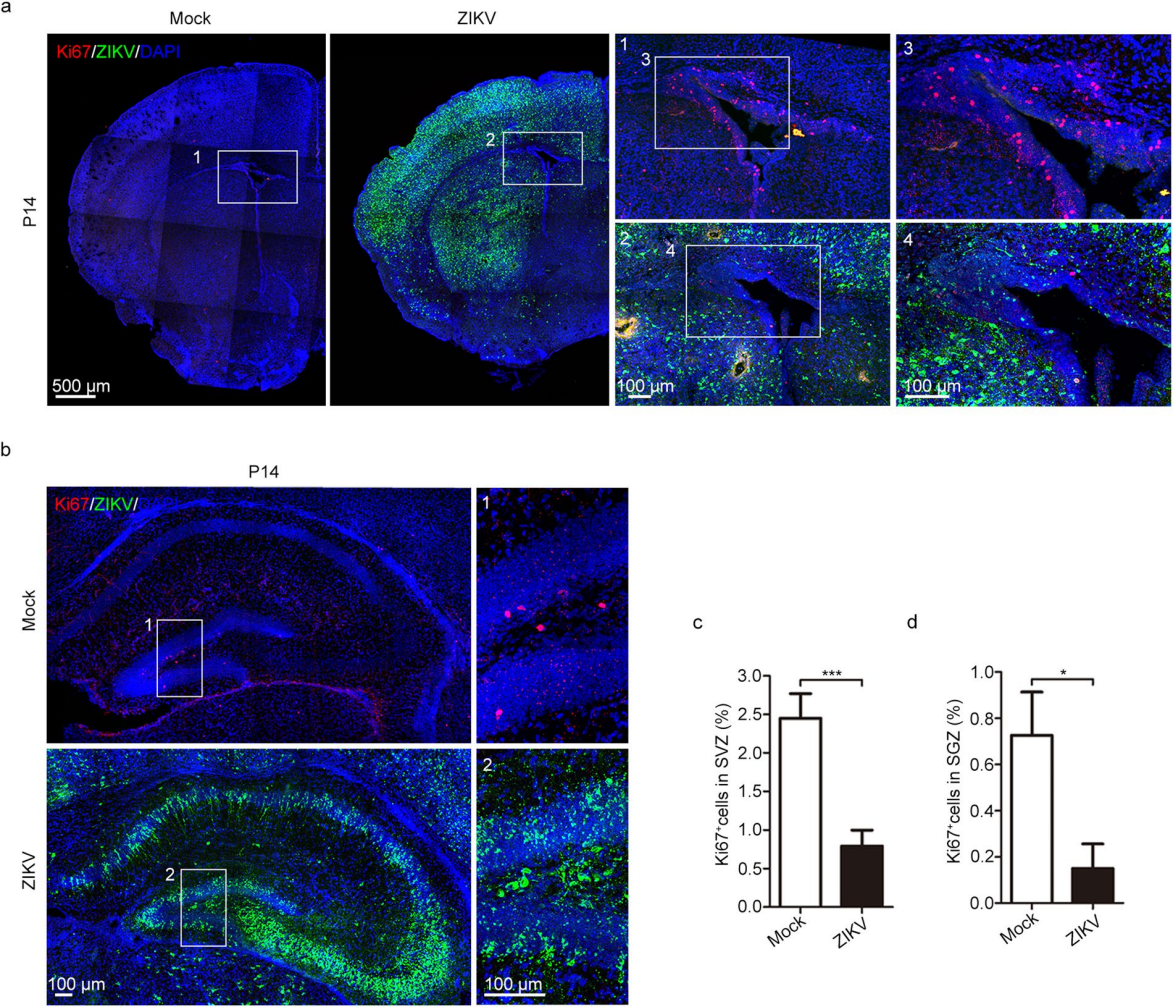
Impaired adult neurogenesis was induced by maternal ZIKV infection. (a) Immunofluorescence staining of Ki67 and ZIKV in the SVZ. (b) Immunofluorescence staining of Ki67 and ZIKV in the SGZ of the hippocampus. (c,d) Percentage of Ki67-positive cells in the SVZ (c) and SGZ (d), respectively. n^Mock^ = 9, n^ZIKV^ = 8 samples from three independent experiments. Data are presented as the mean ± SEM. Student’s *t*-test, *p < 0.05, ***p < 0.001.

In the adult brain, neurogenesis has been demonstrated in several mammalian species, including rodents and primates^15,41^. Adult neurogenesis mainly occurs in two regions: the subventricular zone (SVZ) located in the walls of the lateral ventricles, where new neurons are generated and then migrate through the rostral migratory stream (RMS) to the olfactory bulb; the subgranular zone (SGZ) in the dentate gyrus of the hippocampus, where new dentate granule cells are generated^42^. Since ZIKV preferentially infected embryonic neural stem cells^18^, we then investigated the effects of maternal ZIKV infection on the adult neural stem cells of offspring at P14. ZIKV-E-positive signals could be detected in both the SVZ and SGZ of ZIKV-infected P14 offspring (Fig. 6a,b). Active proliferation ability is an important characteristic of adult neural stem cells; thus, proliferation was evaluated via Ki67 immunostaining. Our results showed that Ki67-positive cells in both the SVZ and SGZ were significantly reduced in ZIKV-infected P14 offspring (Fig. 6a–d). Adult neural precursor cells, visualized by Sox2, were also decreased in both the SVZ and SGZ of ZIKV-infected P14 offspring (Supplementary Fig. S6a–d). This finding indicates that pregnant women suffering from ZIKV infection may have children with impaired adult neurogenesis. Other than Sox2, glial fibrillary acidic protein (GFAP) is also classically considered as a marker of adult neurogenic stem cells in both the SVZ and SGZ^43^. Interestingly, we observed increased GFAP-positive cells in these regions of the P14 ZIKV-infected offspring (Supplementary Fig. S6e–f). Since GFAP is also a marker of astrocytes that participate in the neural immune response, this increase in GFAP-positive cells may result from an enhanced immune response to ZIKV infection.

## Discussion

**Figure 7:**
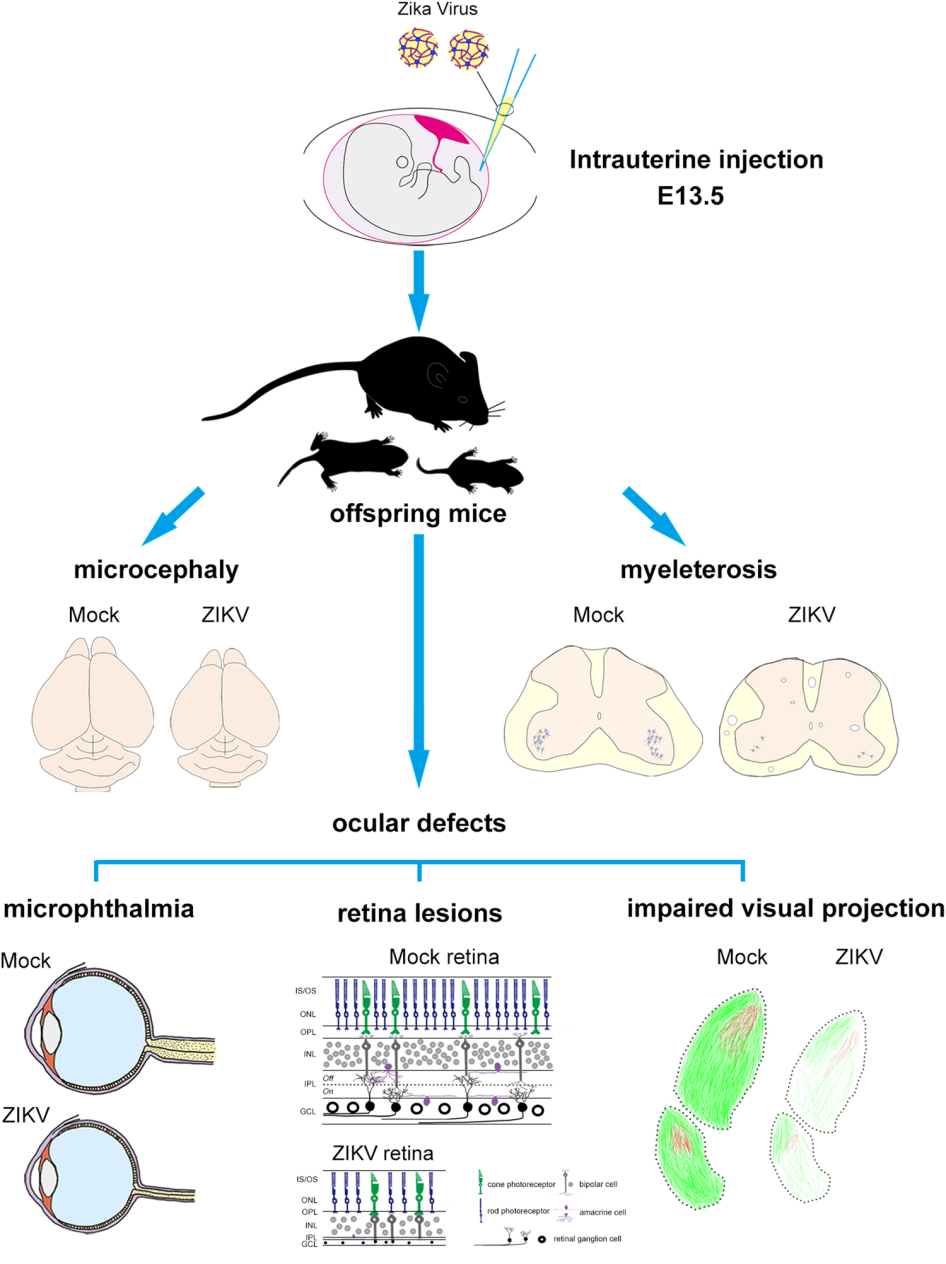
Schematic summary of offspring pathological phenotypes caused by intrauterine ZIKV inoculation.

In pregnant women, ZIKV infection causes fetal demise, IUGR, microcephaly, and eye abnormalities^7^. These syndromes indicate the broad neurological dysfunction in the babies of ZIKV-infected, pregnant women. Several rodent and non-human primate animal models have been used to explore ZIKV pathogenesis, related mechanisms, clinical treatments, and vaccines^18,25–29,44,45^. To evaluate the relationship between ZIKV congenital infection and defects in embryonic and neonatal development, we established a mouse model in which pregnant C57BL/6 mice were intrauterinely infected with ZIKV. Given their intact immune system, C57BL/6 mice more closely mimic the viral infection conditions of humans. Thus, this model may be better for exploring the mechanism of natural ZIKV vertical transmission and how ZIKV impacts neonate development. In humans, ZIKV could be detected in the placenta and amniotic fluid of pregnant women with microcephalic fetuses^9,10^. After intrauterine inoculation of ZIKV into the amniotic fluid of pregnant mice, we observed robust ZIKV infection and consequent apoptosis in the cerebra, cerebella, spinal cords, and eyes of the offspring. Consequently, the offspring of the ZIKV-infected mothers showed a low birth rate, microcephaly, eye abnormalities, paralysis, and early death (Fig. 7), which coincided with epidemiology reports in humans^46^. Interestingly, apart from the cerebral cortex, severe ZIKV infection and apoptosis were also observed in the hippocampus and amygdala. This finding suggests that neonates subjected to maternal ZIKV infection during pregnancy may have problems with memory^47^, orientation^48^ and emotion^49^ as they mature. Along with reduced number, the impaired proliferative ability of adult neural stem cells located in the SVZ and SGZ of offspring was also confirmed. These data suggest that ZIKV impairs adult neurogenesis. Adult neurogenesis is thought to play an important role in learning and memory, emotion, stress, and depression^50^. Defective adult neurogenesis may further imply memory and emotional troubles in the neonates of ZIKV-infected mothers. These issues have not been observed in ZIKV-infected, human newborns because of their young age. However, future studies are required to investigate these ZIKV outcomes.

Eye abnormalities, such as microphthalmia, retinal pigmentary changes, chorioretinal atrophy, vasculature changes, and optic nerve hypoplasia, were reported in fetuses and newborns with vertical ZIKV infection^19–23^. However, the detailed pathological changes were not clear. In our system, ZIKV infection led to smaller eyeballs and thinner optic nerves in postnatal mice. In addition, thinner retinal GCL, IPL, INL, and ONL, as well as an absent OPL were found. Thus, the retinae of the ZIKV-infected offspring were extremely thin. It is known that synapses between the photoreceptor cells and bipolar cells are located at the OPL. Meanwhile, synapses between the bipolar cells and ganglion cells, as well as synapses between the amacrine cells and ganglion cells, are located at the IPL^38^. The missing OPL and thinned IPL reveal visual neural connection defects. In addition, ZIKV infection depleted the majority of ganglion cells in the GCL, which may account for the pathogeny of GCL and optic nerve defects. The optic nerve defects impaired visual projection in different brain areas.

Starburst amacrine cells receive input from bipolar cells and, in turn, make synaptic connections with direction selective ganglion cells (DSGCs). Starburst amacrine cells play an essential role in directionally selective retinal circuits^39^. The complete depletion of starburst amacrine cells also suggests visual defects in ZIKV-infected mouse infants. We found that the ZIKV-induced visual pathological changes progressed during development as more severe symptoms were found in P14 compared to P7 mice. Consistent with our observations, severe visual defects were reported in human babies aged between 4 to 7 months^51^. The visual defects that occurred in the infants of ZIKV-infected mothers will seriously impact their daily activities. Although some anatomical and visual projection differences exist between humans and rodents, our model system is still valuable for exploring the pathological changes and clinical treatments of the visual system.

Based on *in vitro* studies in 293 T cells, keratinocytes, and endothelial cells, one member of the TAM receptor family, AXL, has been suggested to function as an attachment or entry factor for ZIKV^35–37^. Using single-cell RNA-seq and immunohistochemistry, Nowakowski, *et al*. confirmed that AXL is highly enriched in the neural stem cells of the human fetal cerebral cortex, as well as *in vitro*-derived progenitor cells^34^. Based on these studies, AXL is speculated to be a candidate ZIKV attachment or entry factor. In our study, the AXL expression pattern in the retinae of P7 mice is coincident with the retinal cell tropism of ZIKV infection, which is in line with Nowakowski’s study. However, Hastings, *et al*. recently reported that TAM receptors are not required for ZIKV infection in mice^52^. Therefore, further studies are needed to explore the precise mechanism of ZIKV infection.

Hind limb paralysis was reported in ZIKV-infected neonatal and adult mice^27,44^. In addition, arthrogryposis, muscle atrophy, and akinesia were observed in infants born from ZIKV-infected mothers^46^. In our model system, vertical transmission of ZIKV resulted in a loss of alpha motor neurons, thinning of the spinal cords, and malformation of the cerebella. Alpha motor neurons are large, multipolar lower motor neurons of the spinal cord. While the alpha motor neurons are responsible for muscle contraction, the cerebellum plays an important role in movement control. Hence, our rodent model, to some extent, explains the pathogeny of paralysis caused by ZIKV infection and elucidates the movement deficiencies in humans.

DENV and ZIKV are positive-stranded, RNA viruses transmitted via the same vector and are both members of the *Flaviviridae* family^53^. Due to these similarities, DENV-2 was used in our study to test whether it could trigger analogous ZIKV symptoms via our established inoculation routes. However, maternal intrauterine inoculation could not induce valid DENV-2 infection in the offspring. In a few animals, some tissues only had tiny amounts of detectable DENV-2 RNAs at P7, but at P14 these RNAs became undetectable. Furthermore, no DENV-2 E protein could be visualized by immunofluorescence staining at either P7 or P14. Importantly, no ZIKV-induced developmental abnormalities, such as microcephaly, visual defects, and paralysis, were observed in DENV-2-infected offspring. Thus, the neurological defects are specific to ZIKV infection. Other research groups have used DENV in their studies, and ZIKV-specific results were observed as well^17,29^. ZIKV induces placental infection and damage, which boosts the ability of ZIKV to cross the placental barrier^26,28^. Thus, ZIKV may possibly adapt its genome to promote placental infection. This may explain why DENV does not have strong congenital teratogenicity. ZIKV mainly targets the neural system, placenta, ocular tissues, and reproductive system^30^. Therefore, aside from the neural system, our model can also be adopted to study biological disorders in other tissues or organs. This model expands the existing *in vitro* and *in vivo* ZIKV infection models and provides a new platform for unveiling the possible congenital diseases caused by ZIKV. Furthermore, the model can be used to screen for candidate vaccines and therapies.

## Materials and Methods

### Cells, ZIKV antibody and viruses

African green monkey kidney epithelial cells (Vero, ATCC-CCL-81) and baby hamster kidney cells (BHK-21, ATCC-CCL-10) were maintained in DMEM (Invitrogen, US) supplemented with 10% fetal bovine serum (FBS) at 37 °C with 5% CO_2_. Anti-ZIKV human mAb Z6 were generated from activated plasma blasts of hospitalized patients. The sequences encoding heavy and light chains from single-cell cDNA were amplified and cloned into expression vectors, and recombinant mAb was produced via transfection into 293 T human embryonic kidney cells^31^. ZIKV strain ZIKA-SMGC-1 (GenBank accession number: KX266255) was isolated previously and DENV-2 (strain 43, GenBank accession number: AF204178) was a kind gift from Prof. Chengfeng Qin from Beijing Institute of Microbiology and Epidemiology, Beijing, China. Virus stocks were propagated in Vero cells and titrated in BHK-21 cells by standard plaque forming assay.

### Animal

C57BL/6 (WT) female mice were purchased from the Beijing Vital River Laboratory Animal Technology and subsequently bred and housed at Institute of Microbiology, Chinese Academy of Sciences (IMCAS). Pregnancy studies were timed by the presence of a plug, indicating gestational age E0.5. All animal handling procedures were performed in compliance with the PR China Legislation for the Care and Use of Laboratory Animals. The experiments and protocols were approved by the Committee on the Ethics of Animal Experiments of IMCAS, permit number APIMCAS2017006. All animal experiments were conducted under isoflurane anesthesia to minimize animal suffering. Studies with ZIKV were conducted under biosafety level 2 (BSL2) and animal BSL3 (A-BSL3) containment.

### Intrauterine inoculation

At E13.5, C57BL/6 pregnant mice were anesthetized continuously with isoflurane and underwent a mini-laparotomy in the lower abdomen to expose the uterine horns. Animals were randomly assigned to receive either DMEM, ZIKV or DENV-2 inoculation. ZIKV, DENV-2 or DMEM were directly injected into amniotic fluid at the dose of 1500 PFU/fetus. The uterine horns were put back into the abdominal cavity to allow the embryos to continue normal development. Routine closure was performed after injections and dams were returned to individual cages for recovery.

### Quantitation of ZIKV and DENV burden in mice

To detect viral RNA in tissue, all tissues were weighed and homogenized with zirconia beads in a MagNA Lyser instrument (Roche Life Science) with 500 µL ice-cold PBS. All homogenized tissues of infected mice were stored at −80 °C until virus titration. Viral RNAs were extracted with RNeasy Mini Kit (tissues). Using One Step PrimeScript RT-PCR kit (TaKaRa, Japan), real-time qRT-PCR was adopted to determine RNA levels via an ABI 7500 fast Instrument. Viral burden was expressed on a log10 scale as viral RNA copies per g after comparison with a standard curve. The real-time PCR primers set for ZIKV RNA detection was: forward, 5′-TGAYAAGCARTCAGACAC-3′ and reverse, 5′-TCACCARRCTCCCTTTGC-3′. For DENV-2, the primer was: forward, 5′-CAGGCTATGGCACTGTCACGAT-3′ and reverse, 5′-CCATTTGCAGCAACACCATCTC-3′. Real-time qRT-PCR experiments were performed from three independent RNA preparations.

### Immunohistochemistry and antibodies

Animals were transcardially perfused with ice-cold PBS (pH 7.4) followed by 4% paraformaldehyde in PBS (pH 7.4) at different postnatal stages, then brains and eyeballs were dissected out and post fixed in 4% paraformaldehyde overnight at 4 °C. After washing in PBS for 1 h, the samples were immersed in 30% sucrose in PBS until saturation, followed by cryosection at 40μm thickness. The sections were washed by PBS for 3 times and blocked with blocking buffer (10% DS, 0.1% Triton, 1 × PBS) at room temperature for 2 hours. The sections were then incubated with the primary antibody at 4 °C overnight. The antibodies used for immunostaining were activated-Caspase3 (CST, 9664 s, 1:500), AXL (Santa Cruz, SC-1096, 1:200), Calretinin (Millipore, AB1550, 1:200), ChAT (Millipore, AB144P, 1:200), Sox2 (Santa Cruze, sc-17320, 1:200), Ki67 (Millipore, AB9260, 1:200), GFAP (CST,3670 s, 1:500) and Z6^31^ (2 μg/ml). After rinsing with 0.1%PBST, secondary antibody was incubated for 2 hours at room temperature. Finally, after sections were washed with 0.1%PBST, nuclei were stained with 4′, 6-diamidino-2-phenylindole (DAPI) (Invitrogen,1:500).

### Nissl staining

After stepwise hydration (100% alcohol 3 minutes × 2, 95% alcohol 3 minutes, 75% 3 minutes, 50% alcohol 3 minutes, distilled water 30 s), spinal cord slices were stained with 0.1% Cresyl Violet acetate for 6 mins and dehydrated in turn by distilled water (30 s), 50% ethanol (1 minute), 75% (2 minutes), 95% ethanol (2 minutes), 100% alcohol (3 minutes) × 2. Finally, slices were hyalinized by Xylene.

### Confocal imaging

Slices were imaged on a confocal microscope (Olympus FluoView™ FV1000), and the images were analyzed with Imaris, ImageJ, and Photoshop.

### Intraocular injection

We adopted procedures previously described for intraocular injection^54,55^. At P14, mice were anesthetized with isoflurane and put under a dissecting microscope to ensure clear visibility of the tips of glass micropipettes. Penetrating the cornea by inserting glass micropipettes into the juncture of cornea and sclera, 1 µl of 1 mg/ml CTB-488(Invitrogen, C34775) and 1 µl of 1 mg/ml CTB-594 (Invitrogen, C34777) were injected into either eye. To avoid leakage, each intraocular injection persisted at least one minute and micropipette held for one extra minute before withdrawal.

### Visualization of visual projection

After intraocular injection, animals were sacrificed 3 days later and intracardially perfused with 4% paraformaldehyde (PFA) in PBS. The brains and eyeballs were postfixed overnight in 4% PFA. After washed in PBS, coronal sections were cut at 150 µm on a vibratome (Leica, VT1200S). Images were captured using a confocal microscope (Olympus FluoView™ FV1200). Mean gray value of visual projection fibers was measured by using ImageJ 1.42.

### Quantification and statistical analysis

All data were analyzed for statistical significance with the SPSS17.0 Package (SPSS, US). All numerical data are presented as the mean ± SEM (standard error of mean). p values less than 0.05 were considered significant. All graphs were generated with Graph Pad Prism 5.0 (Graph Pad Software, US).

## Electronic supplementary material

Supplementary figures and figure legends

Movie S1

Movie S2

Movie S3
