# Supplementary material for: Vertical Transmission of the Zika Virus Causes Neurological Disorders in Mouse Offspring
Source: Sci Rep. 2018 Feb 23;8:3541. doi: 10.1038/s41598-018-21894-w (PMC5824946; doi:10.1038/s41598-018-21894-w)
Supplement: Supplementary file 1 — Supplementary figures and figure legends [file 41598_2018_21894_MOESM1_ESM.pdf]

Vertical Transmission of the Zika Virus Causes Neurological Disorders in Mouse Offspring

Yingchao Shi[1,3,#], Shihua Li[2,#], Qian Wu[1,3,#], Le Sun[1,3], Junjing Zhang[1], Na Pan[1,3], Qihui Wang[2], Yuhai Bi[2], Jing An[4,7], Xuancheng Lu[9], George Fu Gao[2,5,6,8,*] and Xiaoqun Wang[1,3,4,*]

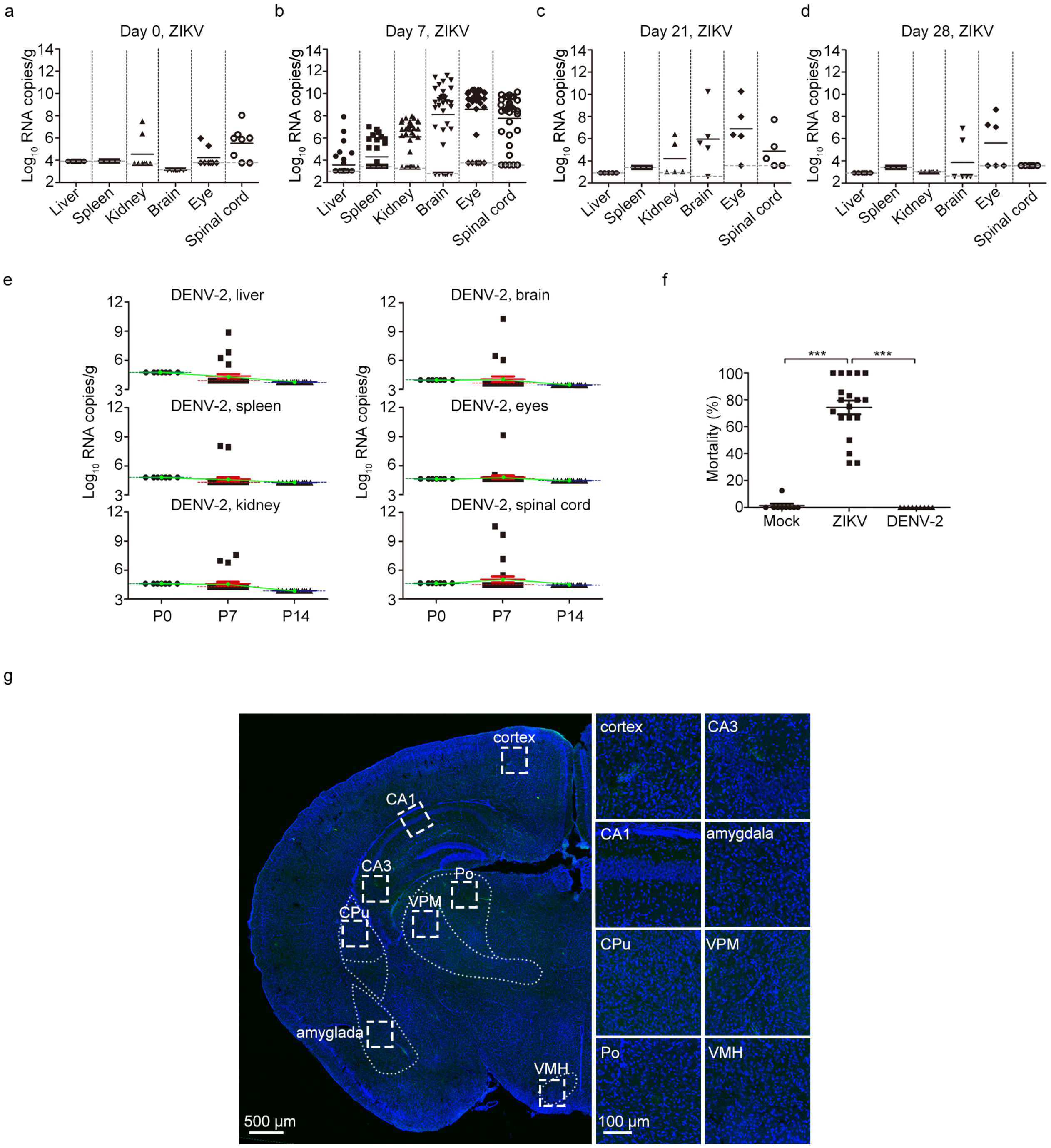

Supplementary Figure 1

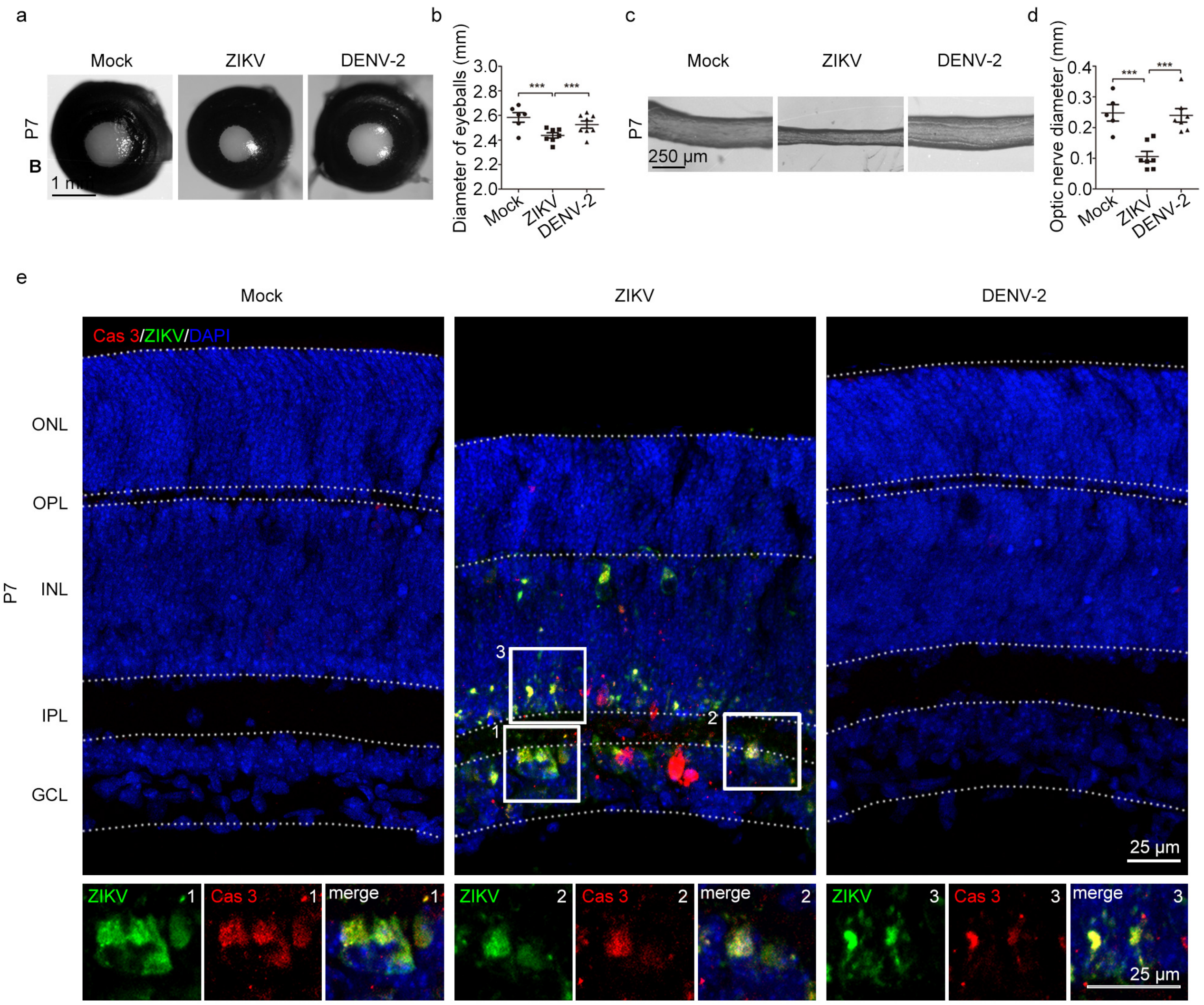

Supplementary Figure 2

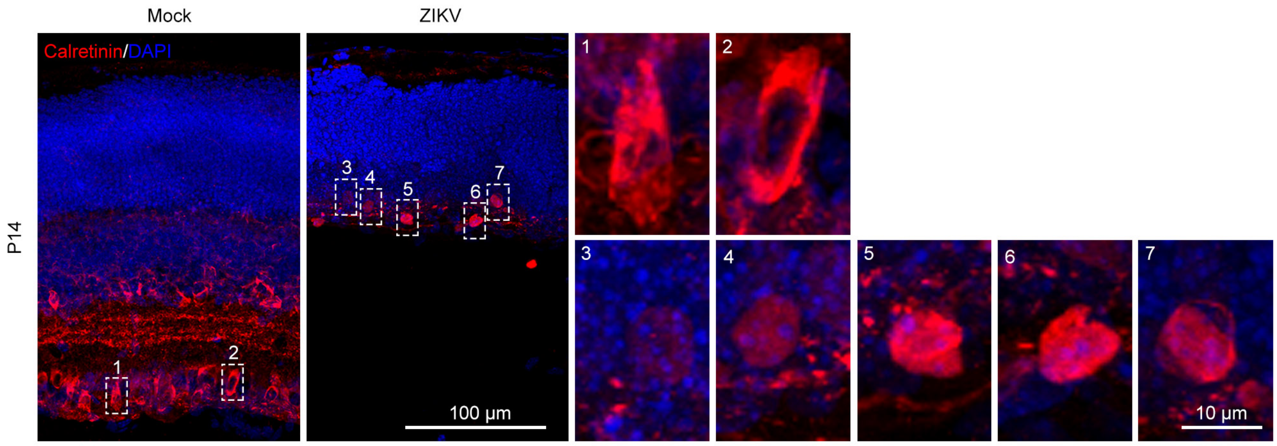

Supplementary Figure 3

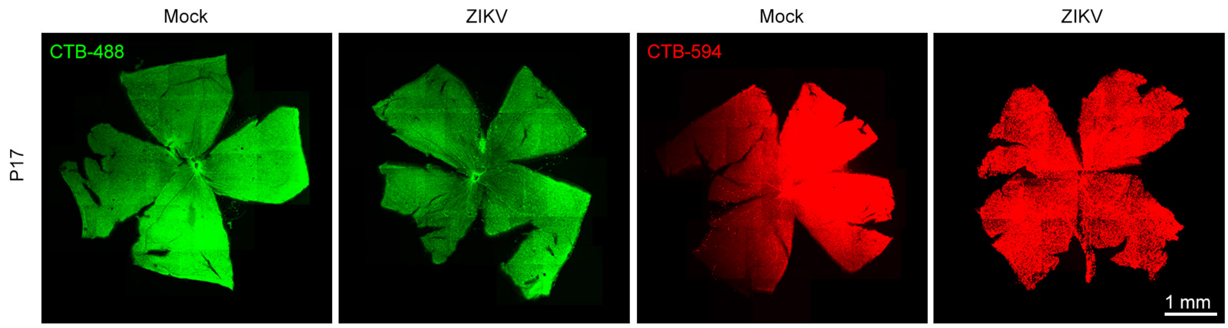

Supplementary Figure 4

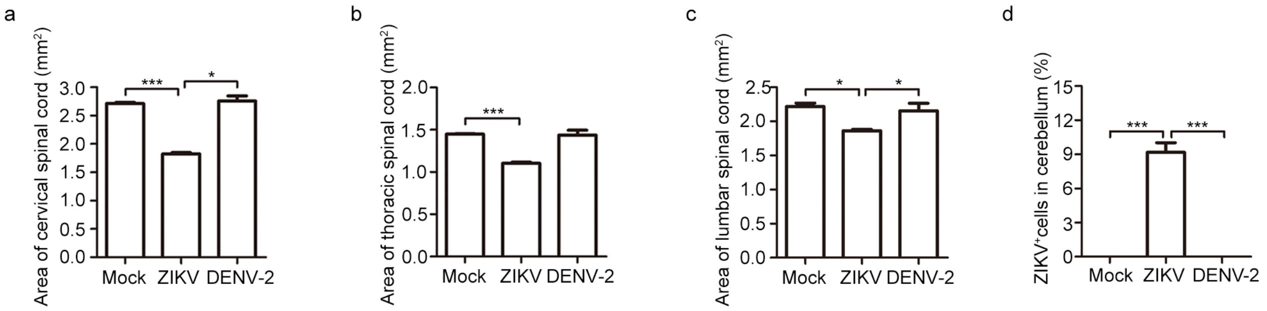

Supplementary Figure 5

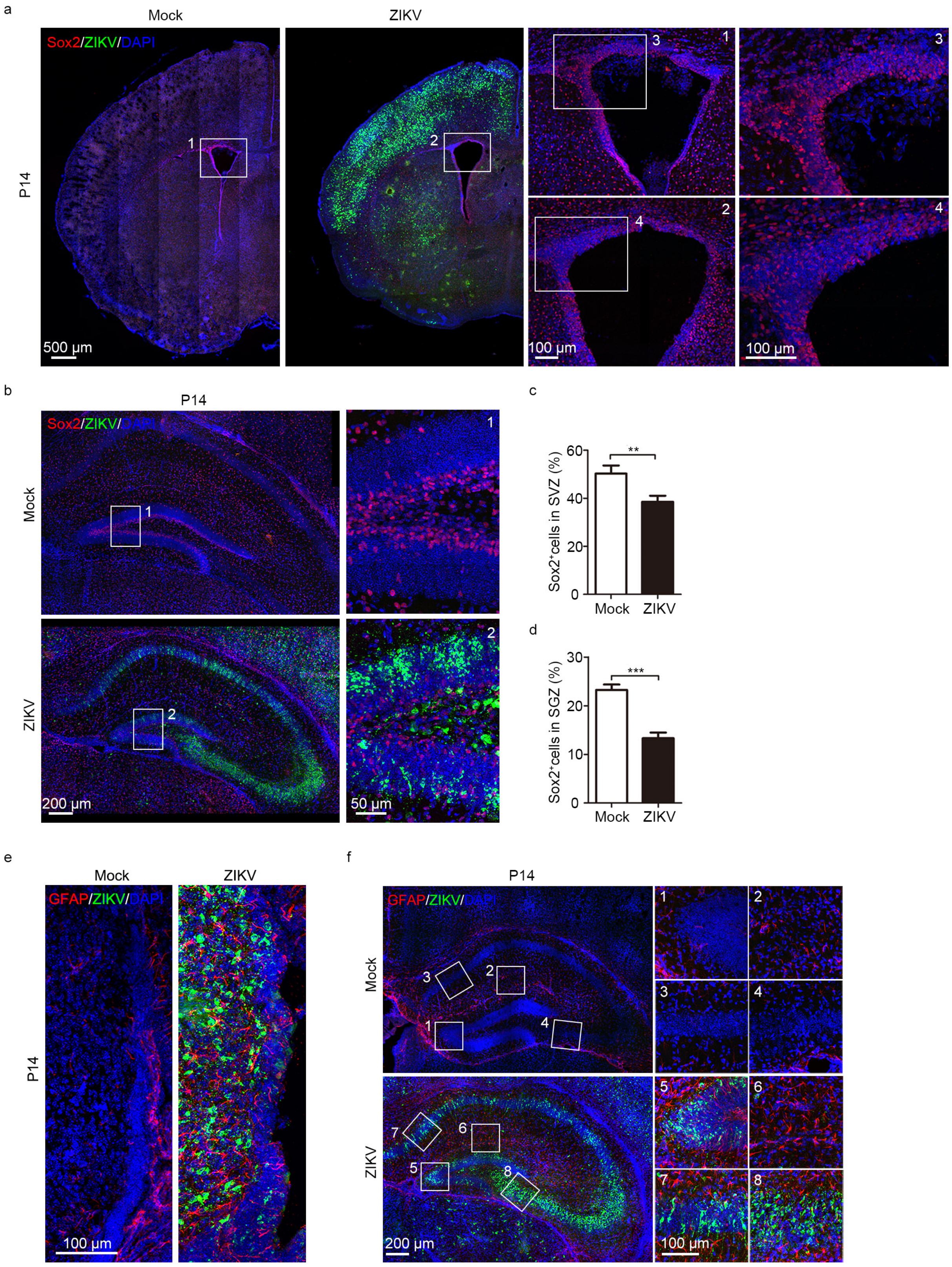

Supplementary Figure 6

Supplementary information

Figure Legends

Figure S1 Virus infection in postnatal mice.

(a-d) ZIKV viral load in tissues at P0(a), P7(b), P21(c), and P28(d) was measured by qRT-PCR. Bars indicate the mean of the data. The gray dashed lines indicate the limit of detection (e) DENV-2 viral load in tissues at P0, P7, and P14 was measured by qRT-PCR. Bars indicate the mean ± SEM of the data. The dashed lines indicate the limit of detection. The change tendency of the viral load is indicated by the green lines. (f) Mortality of offspring mice during P0-P28. Data are presented as the mean ± SEM. One-way ANOVA with Bonferroni's post hoc test, ***$p< 0.001$. (g) Overview of DENV-2 infection in 1 brain slice. CPu, caudate putamen (striatum); Po, posterior thalamic nuclear group; VPM, ventral posteromedial thalamic nucleus; VMH, ventromedial hypothalamic nucleus.

Figure S2 Visual defects were detected at postnatal day 7.

(a) Representative images of P7 eyeballs. (b) Diameters of P7 eyeballs. (c) Representative images of P7 optic nerves. (d) Diameters of P7 optic nerves. b and d, data are presented as the mean ± SEM; One-way ANOVA with Bonferroni's post hoc test, ***$p < 0.001$. (e) Representative images of P7 retinae stained for CAS3 and ZIKV. The areas outlined by a white box are magnified in the lower panels.

FigureS3 Abnormal retinal Calretinin-positive cells in ZIKV-infected mice. Retinal Calretinin immunostaining in P14 offspring mice. The areas outlined by a white box are magnified in the panels on the right.

Figure S4 Retinae were homogeneously colored by intraocular injection of CTB-488 or CTB-594. Images of homogeneously stained retinae after intraocular injection of CTB-488 or CTB-594.

Figure S5 ZIKV infection causes pathological changes in spinal cords and cerebella.

(a-c) Quantification of the area of transections of different spinal cord segments. n=5

samples from three independent experiments. (d) Percentage of ZIKV-infected cells in the cerebellum. n=3 samples from three independent experiments. Data are presented as the mean ± SEM. One-way ANOVA with Bonferroni's post hoc test, *$p < 0.05$, ***$p < 0.001$.

Figure S6 Maternal ZIKV infection reduced neural stem cells in the SVZ and SGZ. (a) Immunofluorescence staining of Sox2 and ZIKV in the SVZ. The areas outlined by a white box are magnified in the middle and right panels. (b) Immunofluorescence staining of Sox2 and ZIKV in the SGZ. (c-d) Percentage of Sox2-positive cells in the SVZ(c) and SGZ(d), respectively. (c) $n^{Mock}=3$, $n^{ZIKV}=4$ samples from three independent experiments. (d) n=5 samples from three independent experiments. Data are presented as the mean ± SEM; Student's t-test, **$p < 0.01$, ***$p < 0.001$. (e-f) Immunofluorescence staining of GFAP and ZIKV in the SVZ (e) and SGZ (f), respectively.

Movie S1. Movement of a wild-type mouse at P14.

Movie S2. Movement of a ZIKV-infected mouse at P14.

Movie S3. Movement of a DENV2-infected mouse at P14.
